# Photoperiod Mediated Changes in Olfactory Bulb Neurogenesis and Olfactory Behavior in Male White-Footed Mice (*Peromyscus leucopus*)

**DOI:** 10.1371/journal.pone.0042743

**Published:** 2012-08-09

**Authors:** James C. Walton, Leah M. Pyter, Zachary M. Weil, Randy J. Nelson

**Affiliations:** Department of Neuroscience, The Ohio State University Wexner Medical Center, Columbus, Ohio, United States of America; Morehouse School of Medicine, United States of America

## Abstract

Brain plasticity, in relation to new adult mammalian neurons generated in the subgranular zone of the hippocampus, has been well described. However, the functional outcome of new adult olfactory neurons born in the subventricular zone of the lateral ventricles is not clearly defined, as manipulating neurogenesis through various methods has given inconsistent and conflicting results in lab mice. Several small rodent species, including *Peromyscus leucopus*, display seasonal (photoperiodic) brain plasticity in brain volume, hippocampal function, and hippocampus-dependent behaviors; plasticity in the olfactory system of photoperiodic rodents remains largely uninvestigated. We exposed adult male *P. leucopus* to long day lengths (LD) and short day lengths (SD) for 10 to 15 weeks and then examined olfactory bulb cell proliferation and survival using the thymidine analog BrdU, olfactory bulb granule cell morphology using Golgi-Cox staining, and behavioral investigation of same-sex conspecific urine. SD mice did not differ from LD counterparts in granular cell morphology of the dendrites or in dendritic spine density. Although there were no differences due to photoperiod in habituation to water odor, SD mice rapidly habituated to male urine, whereas LD mice did not. In addition, short day induced changes in olfactory behavior were associated with increased neurogenesis in the caudal plexiform and granule cell layers of the olfactory bulb, an area known to preferentially respond to water-soluble odorants. Taken together, these data demonstrate that photoperiod, without altering olfactory bulb neuronal morphology, alters olfactory bulb neurogenesis and olfactory behavior in *Peromyscus leucopus*.

## Introduction

Neurogenesis in discrete areas of the mammalian brain is an ongoing process which continues throughout adulthood. Nascent granule neurons arise in the subgranular zone (SGZ) of the hippocampus and are incorporated into the dentate gyrus as they mature. Neural progenitor cells arising from the subventricular zone (SVZ) of the lateral ventricles migrate via the rostral migratory stream (RMS) to the olfactory bulbs. Once in the olfactory bulb (OB), they migrate radially outward and mature; mainly becoming interneurons (reviewed in [1]). Although the function of adult neurogenesis in the hippocampus has been well described [2], the behavioral and functional outcomes of adult neurogenesis within the olfactory bulb remain unspecified (reviewed in [1], [3]).

Olfactory information enters the central nervous system through the sensory olfactory epithelium in the nasal cavities (and the vomeronasal organ in many species), and is then relayed through the olfactory nerve to the glomerular layer of the olfactory bulb, synapsing on the tufted and mitral cells, which are the projection neurons of the olfactory bulb. In addition to forming synaptic connections with the tufted and mitral cells of the olfactory bulb, the inhibitory interneurons of the granule cell layer and periglomerular cells also receive input from sensory neurons in the olfactory epithelium [4]. In adult mammals, the granule and periglomerular cells, which modulate the projection cells from the olfactory bulb, are continuously replaced via neurogenesis [5]. Continuous adult neurogenesis is necessary to maintain innate olfactory responses [6] and it has been recently hypothesized that the continuous turnover of the inhibitory granule cells in adulthood is responsible for optimizing pattern separation, thus optimizing encoding of olfactory information [7].

In a naturalistic context, optimization of olfactory information is critical for fitness, especially in animals that rely primarily on olfactory information for social communication and predator avoidance, such as small rodents. These social and avoidance cues can vary seasonally [8]–[10]. Photoperiodism is the biological ability of animals to track day length and to make seasonally appropriate adaptive responses to survive differing seasonal energetic demands in non-tropical latitudes (reviewed in [11]). Photoperiodic changes in olfaction and odor responsiveness, which are generally associated with reproduction, have been identified in many species across vertebrate and invertebrate taxa (reviewed in [12]). Seasonal changes in hippocampal neurogenesis have been documented in several photoperiodic rodent species [13]–[17]; however, few studies have investigated the role of photoperiod on SVZ/OB neurogenesis in these species [15], [17], and to our knowledge, no studies have demonstrated a functional difference in olfaction associated with photoperiodic changes in olfactory bulb neurogenesis in rodents.

White-footed mice (*Peromyscus leucopus*) are small photoperiodic rodents, indigenous to central and northern regions of the United States east of the Rocky Mountains [18]. As with other well-studied photoperiodic small rodents, the reproductive, immunological, and behavioral responses to photoperiod have been well described in this species [19]–[22]. However, the effects of photoperiod on olfactory bulb neurogenesis and associated olfactory behavior remain undescribed. Toward this end, we assessed the role of photoperiod on olfaction, olfactory bulb neuronal morphology, and olfactory bulb neurogenesis in male white-footed mice by asking the following questions: 1) does photoperiod alter cell proliferation and neurogenesis in the olfactory bulbs of this species, 2) does photoperiod alter olfactory bulb neuronal morphology, and 3) are photoperiod-mediated changes in olfactory bulb neurogenesis or morphology associated with changes in an olfactory behavior?

## Materials and Methods

### Animals

Sixty-five adult (>55 days of age) male white-footed mice (*Peromyscus leucopus*) from our breeding colony were used in this study. Animals were housed individually in polypropylene cages (27.8×7.5×13 cm) with a constant temperature and humidity of 21±5°C and 50±10%, respectively, and *ad libitum* access to food (Harlan Teklad 8640 rodent diet, Indianapolis, IN) and filtered tap water. Mice were either housed in reversed long days (LD; 16 h light/day), or in short days (SD; 8 h light/day) for 10–15 weeks, depending on experiment. Photoperiodic responsiveness (SD-induced reduction of reproductive tissue mass) was verified for mice in all experiments. All procedures were approved by the Ohio State University Institutional Animal Care and Use Committee and comply with guidelines established by the National Institutes of Health [23].

**Figure 1:**
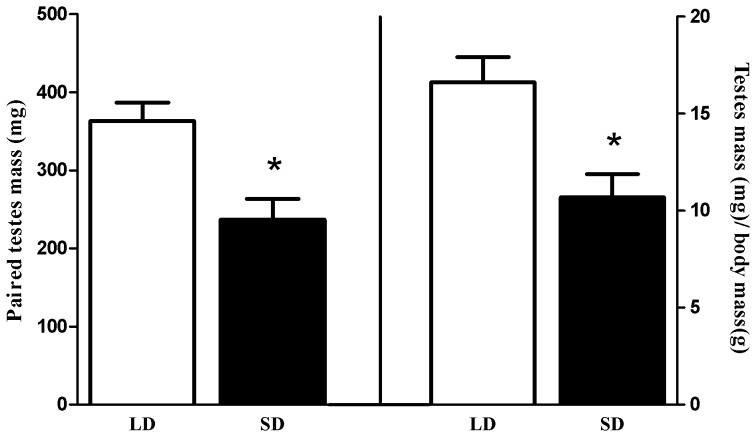
Reproductive responses to photoperiod. Exposure to short day lengths for 10–15 weeks reduced absolute paired testes mass (left) and paired testes mass controlling for body mass differences (right). *p≤0.05.

### Experiment 1: Effects of Photoperiod on Olfactory Bulb Cell Proliferation and Neurogenesis

#### BrdU injections

To estimate neurogenesis, after 10 weeks of photoperiod exposure, 17 mice (n = 11 LD, n = 6 SD) were given daily IP BrdU injections (50 mg/kg in 0.1 ml saline; Sigma-Aldrich, St. Louis, MO, USA) for 6 consecutive days. Prior to perfusions, mice remained undisturbed for 4 weeks following the conclusion of injections. The time of injections was randomized each day to control for circadian differences in cell division among the treatment groups. To estimate olfactory bulb cell proliferation, after 14 weeks in photoperiod, 17 mice (n = 9 LD, n = 8 SD) were given a single intraperitoneal (IP) injection of the cell division marker, bromodeoxyuridine (BrdU 50 mg/kg in 0.1 ml saline) 1.5–2.5 h prior to perfusion.

#### Tissue collection and histology

Following BrdU treatment (proliferation, 1.5–2.5 h; neurogenesis, 4 wk), mice were deeply anesthetized with sodium pentobarbital (Abbott Laboratories, North Chicago, IL, USA) and transcardially perfused with 50 ml of ice-cold saline followed by 75 ml of 4% paraformaldehyde in 0.1 M PBS. Paired testes were removed and weighed to determine reproductive responsiveness to photoperiod treatment. Brains were removed, post-fixed in paraformaldehyde for 3 h at room temperature, transferred into 0.2 M phosphate buffer overnight at 4°C. The next day, brains were transferred to 30% sucrose in 0.1 M PBS until permeated, then frozen and stored at −70°C. Using a cryostat, brains were cut into 25 µm sections and thaw mounted onto positively charged slides (Superfrost Plus, Fisher Scientific, Pittsburgh, PA, USA). For immunohistochemistry, one series of every eighth section from all mice were fluorescently triple-labeled for BrdU, glial fibrillary acidic protein (GFAP), and neuronal nuclei (NeuN) to detect newly-born, glial and neuronal cells, respectively. Briefly, slides were rinsed in 0.1 M TBS for 30 min, incubated in 2N HCl at 37°C for 15 min to denature DNA, and then were immediately transferred to 0.1 Mborate buffer for 10 min at room temperature. Following 3 rinses in TRIS buffered saline (TBS), slides were blocked (3% donkey serum in TBS +0.5% Triton-X +0.2% sodium azide) for 4 h at room temperature with constant agitation. A primary antibody mixture [1∶200 rat anti-BrdU (Accurate Chemical & Scientific Corporation, Westbury, NY, USA), 1∶200 mouse anti-NeuN (Chemicon International, Temecula, CA, USA), 1∶500 rabbit anti-GFAP (Sigma-Aldrich, St. Louis, MO, USA)] was applied to all slides for 24 h at room temperature with constant agitation. Slides were thrice rinsed in TBS and a secondary antibody mixture (1∶200 anti-rat Alexa594, 1∶200 anti-mouse Alex488, 1∶500 anti-rabbit Alexa647, all raised in donkey; Molecular Probes, Carlsbad, CA, USA) was applied to all slides for 3 h at room temperature with constant agitation. Slides were rinsed in TBS and then coverslipped with Fluoromount (Fisher Scientific, Pittsburgh, PA, USA). A mean (±SEM) of 13.9±0.4 sections were analyzed per mouse. BrdU+ cells were manually counted in each section within the periglomerular and plexiform regions of the olfactory bulbs at 400× magnification with a fluorescent microscope. Sections were designated as being rostral (∼300 µm), central (∼500 µm), or caudal (∼750 µm) from the tip of the olfactory bulbs. The number of BrdU+ cells within the granule cell layer was estimated by manually counting the number of cells within 3 (dorsal, medial, ventral) 300 µm×225 µm grid boxes of the granule cell layer at 400× magnification. To determine the cellular phenotypes of BrdU+ cells in the granule cell layer of the neurogenesis brains (i.e., 6 injections of BrdU +4 weeks), between 35–60 cells (mean 46.7±9.3) from 8–15 sections (mean 12±2.7) per animal were analyzed at 400× magnification using a confocal laser scanning microscope (Zeiss 510 META, Thornwood, NY, USA) with excitation wavelengths of 488, 543, and 633 nm at the Microscope and Imaging Facility at Ohio State University.

### Experiment 2: Effects of Photoperiod on Dendritic Morphology in the Olfactory Bulb Granule Cell Layer

#### Olfactory bulb neuronal dendritic morphology

After 10 weeks in respective photoperiods, a separate cohort of mice (n = 5 LD, n = 8 SD) were rapidly decapitated. Brains with complete and intact olfactory bulbs were removed and processed for Golgi staining according to the manufacturer’s protocol (FD Rapid GolgiStain Kit, FD Neurotechnologies, Ellicott City, MD, USA) as previously described [21]. Briefly, olfactory bulbs were cut coronally in 80 µm sections on a cryostat and mounted on 3% gelatin-coated slides, dried for 7–10 days, then counterstained with cresyl violet before dehydration and coverslipping. Granule cells in the olfactory bulbs (n = 5/mouse) were traced using a camera lucida at 400× magnification (Neurolucida, MicroBrightField, Williston, VT, USA). Dendritic spines were traced on five 10 µm distal segments of each neuron at 1000× magnification on the terminal tips of randomly chosen granule cell dendrites that had at least one branch point. Using the accompanying software (NeuroExplorer, MicroBrightField) dendritic complexity (via Sholl analysis), dendritic length, and spine density were calculated. Throughout analyses, all samples were number-coded so the experimenter was unaware of the treatments.

**Figure 2:**
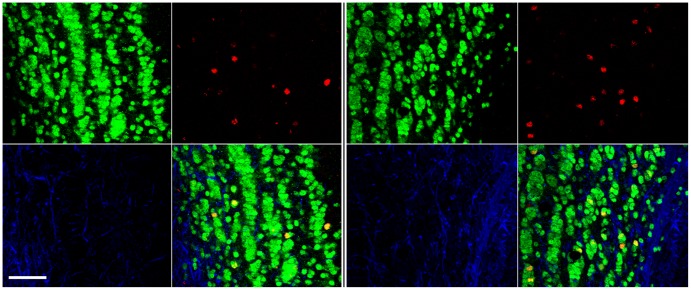
Olfactory bulb photomicrographs from LD (left panel) and SD (right panel) white-footed mice. Within each panel, clockwise from lower left: GFAP (blue), NeuN (green), BrdU (red), and merged images. Scale bar = 50 µm.

**Figure 3:**
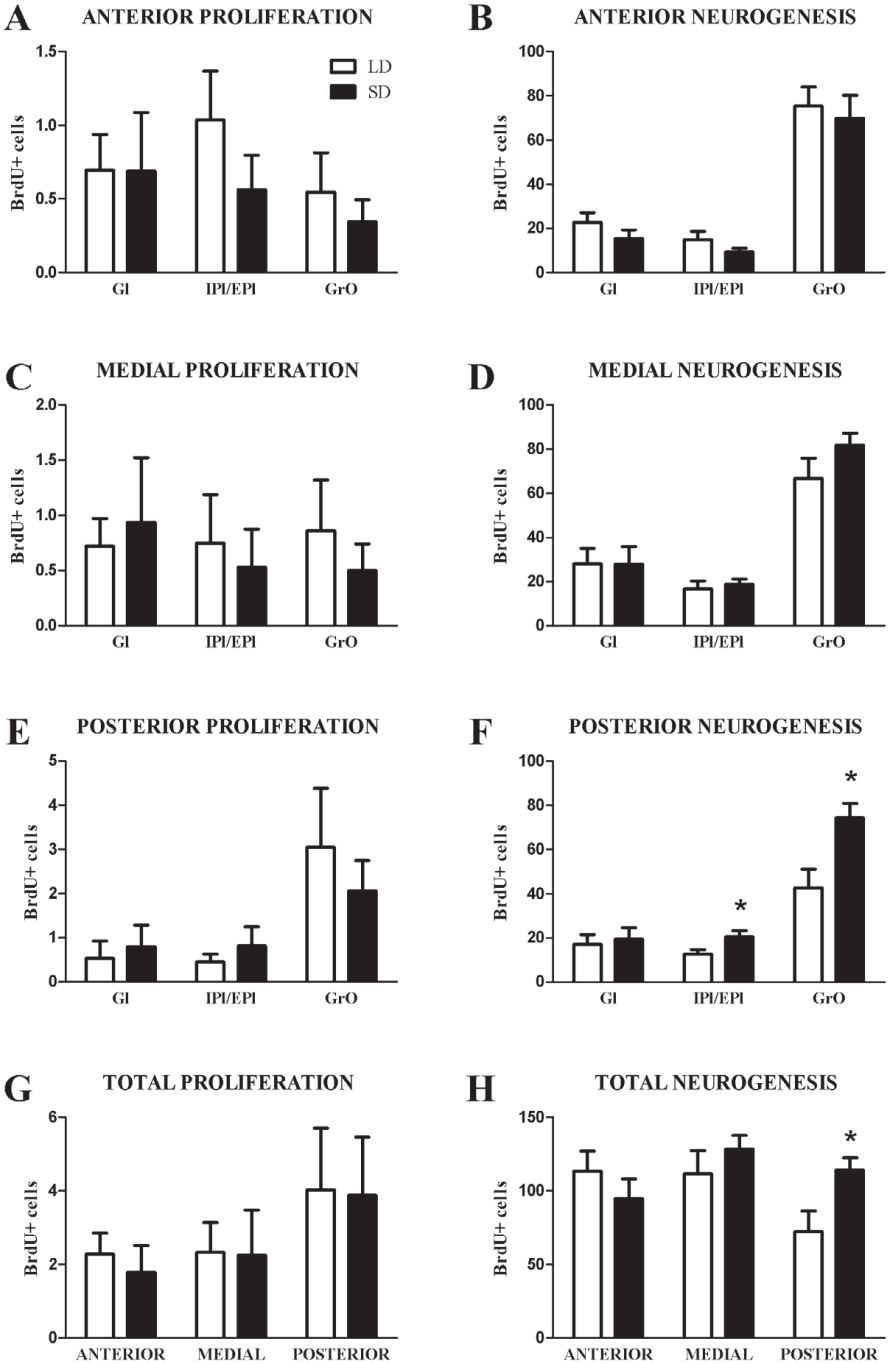
Progenitor cell proliferation and survival in the olfactory bulb. There were no effects due to photoperiod in progenitor cell proliferation 2 h after BrdU injection (A,C,E,G). Exposure to short days increased progenitor survival in the posterior olfactory bulb (H). The majority of these differences were in the posterior plexiform and granule cell layers (F), whereas there were no differences due to photoperiod the medial (D) or anterior (B) olfactory bulb. All abbreviations after Paxinos & Franklin (2004): Gl, glomerular layer of the olfactory bulb; IPl, internal plexiform layer of the olfactory bulb; EPl, external plexiform layer of the olfactory bulb; GrO, granule cell layer of the olfactory bulb. *p≤0.05.

### Experiment 3: Effects of Photoperiod on Investigation of Conspecific Male Urine

#### Habituation-dishabituation test

After 10 weeks of exposure to photoperiod, mice (n = 9 LD, n = 9 SD) were tested under dim red light at the beginning of the dark phase in an olfactory habituation-dishabituation assay. On the first day of the testing paradigm, mice were transferred in their home cages to the behavioral testing room at the onset of the dark phase and allowed to habituate for 1 h, then returned to their respective vivarium rooms. On day 2, mice were transferred to the testing room and allowed to habituate for a minimum of 30 min prior to olfactory testing. For odorants, fresh urine from 6 experimentally naive male *P. leucopus* (3 LD, 3 SD), that were unassociated with the behavioral testing, was collected immediately prior to olfactory testing, pooled in a sterile 1.5 ml micro centrifuge tube, and held on ice along with a fresh aliquot of ddH2O for the duration of testing. All olfactory testing was conducted in one single session using the same odorants for all mice. For odor exposure, the wire food hoppers were removed from their home cages and replaced with an identical sterile empty hopper. Mice were allowed to habituate to the new hopper for 5 minutes, and then a 1000 µl pipette tip containing the scented filter paper was presented 6 times for 3 min with a 1 min interval between presentations. The first 3 presentations were ddH_2_O and the final 3 were male urine. Odorants were presented in the following manner: immediately prior to odor presentation for each mouse, 25 µl of the odorant was placed on a 1 cm^2^ piece of filter paper and inserted into the wide end of a sterile 1000 µl pipette tip 5 mm below the edge, and then the pipette tip was inserted 3 cm, open end down, through the wire cage lid at the front of the cage. The location of the odorant source within the cage was consistent for all mice tested. All behavior was recorded and videos were scored for time spent directly investigating the pipette tip (rearing up and placing snout within 1 cm of the open end of the pipette tip), using The Observer software package (v8.0, Noldus, Leesburg, VA, USA), by an observer unaware of both animal groups and odor treatment.

**Table 1:** Phenotype of progenitor cells 4 weeks after BrdU injections in the granule cell layer of the olfactory bulb of LD and SD exposed male *P. leucopus*.

		% (±SEM) of BrdU+ cells	
	NeuN+	GFAP+	Unlabeled
ALL MICE	99.75	0.25	0.17
LD	99.54±0.46	0.46±1.22	0.86±0.32
SD	100±0.00	0	0

### Statistical Analyses

Olfactory habituation-dishabituation data were analyzed by repeated measures ANOVA, with investigation time as the repeated measure and photoperiod as the between subjects factor. Significant results were followed up by within trial two-tailed Student’s *t*-tests. Two-tailed Student’s *t*-tests were used for comparisons between photoperiods for reproductive responsiveness, dendritic length and spine density, and BrdU measures of cell proliferation and neurogenesis. Repeated measures ANOVAs were used to compare photoperiod effects on dendritic complexity (Sholl analysis). Data with unequal variance were log transformed prior to comparisons. SPSS software (v.19, IBM, Armonk, NY, USA) was used for all analyses, and all comparisons were considered statistically significant if *p*≤0.05 as calculated by SPSS.

**Figure 4:**
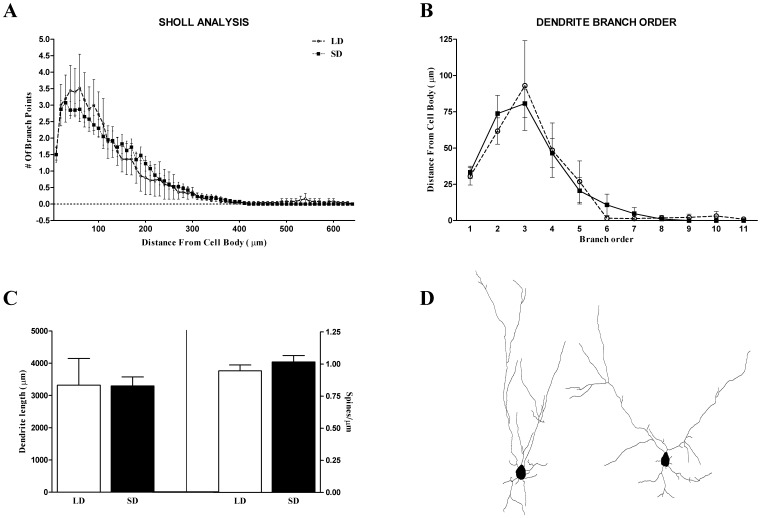
Olfactory bulb neuron morphology. Short day exposure did not alter dendritic intersections (A; Sholl analysis) or branch order of the dendrites (B). There were no differences due to photoperiod in dendrite length (C, left) or spine density (C, right). Representative Neurolucida tracings of olfactory bulb granule cells (D). *p*>0.05 in all cases.

## Results

### Reproductive Responses to Photoperiod

For all mice, exposure to short day lengths for 10–15 weeks reduced paired testes mass (*t*_55_ = 4.508, *p*≤0.05) and paired testes mass corrected for body mass (*t*_53_ = 4.390, *p*≤0.05) (Figure 1).

**Figure 5:**
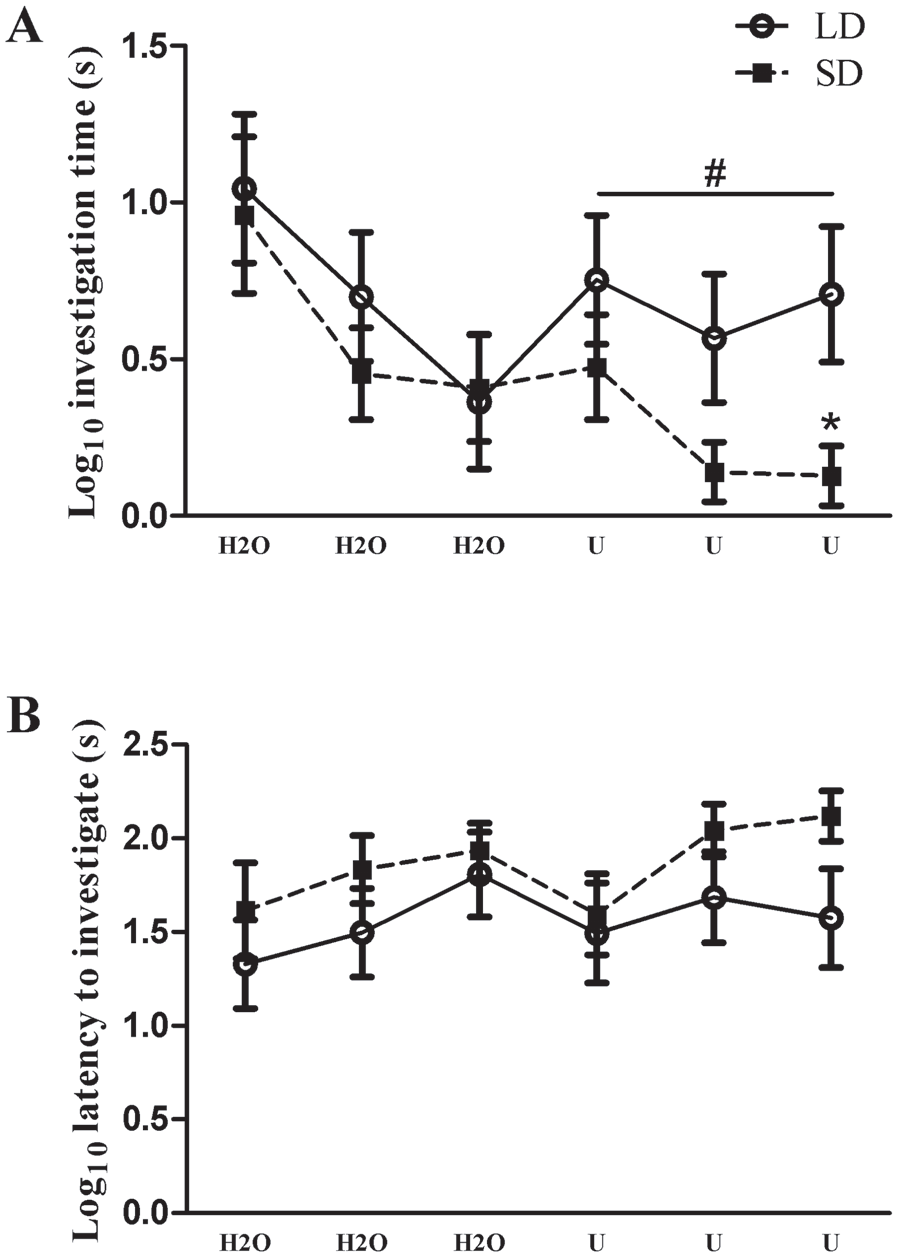
Olfactory habituation-dishabituation test. A) Photoperiod had no effect on habituation to water (H2O), whereas mice exposed to short days spent significantly less time investigating male urine (U, *p*≤0.05 repeated measures ANOVA). SD mice did not differ from LD mice in initial investigation time of novel male urine; however they habituated to the odor faster than their LD counterparts. B) Photoperiod did not alter latency to investigate the presented odor within or across trials. #*p*≤0.05 repeated measures ANOVA, *p≤0.05 Tukey’s HSD.

### Experiment 1

Mice treated to examine neurogenesis (i.e., 6 BrdU injections +4 weeks; Figure 2) displayed more BrdU+ cells in the glomerular layer (Gl), plexiform layers (IPl/EPl), and granular cell layer (GrO) across the rostro-caudal extent of the olfactory bulbs compared with mice treated to examine cell proliferation (i.e., 1 BrdU injection +2 h) (Figure 3). There were no photoperiodic differences in progenitor cell (BrdU+) proliferation or 4 week survival (neurogenesis) in the anterior (*p*>0.05; Figure 3A,B) or the medial olfactory bulb (*p*>0.05; Figure 3C,D). Although there were no photoperiodic differences in proliferation in the posterior olfactory bulbs (*p*>0.05; Figure 3E), exposure to short days increased 4 week progenitor survival in the posterior plexiform (*t*_15_ = −2.256, *p*≤0.05) and posterior granule cell layers (*t*_15_ = −2.524, *p*≤0.05)(Figure 3F). Including all layers across the entire rostro-caudal extent of the olfactory bulbs, there were neither differences due to photoperiod in cell proliferation (*p*>0.05; Figure 3G), nor differences in progenitor survival after 4 weeks in the anterior or medial olfactory bulbs (*p*>0.05; Figure 3F). However, within the posterior olfactory bulb, short days increased 4 week progenitor survival (*t*_15_ = −2.102, *p*≤0.05; Figure 3H).

Within the granule cell layer, the majority of BrdU+ cells were co-labeled with the neuronal marker NeuN, with very sparse GFAP+ or cells labeled with BrdU+ alone (Table 1). No differences in the percentages of different BrdU+ cell phenotypes were observed between photoperiods (Table 1).

### Experiment 2

#### Olfactory bulb granule cell morphology

Exposure to short day lengths did not alter granule cell dendritic complexity in the olfactory bulbs measured by Sholl analysis (F_1,62_ = 0.018; *p* = 0.89; Figure 4A) and branch order analysis (F_1,9_ = 0.000; *p* = 0.99; Figure 4B). Short days did not alter dendritic length (*t*_11_ = 0.033, *p* = 0.97) or dendritic spine density (*t*_11_ = −0.932, *p* = 0.37) (Figure 4C).

### Experiment 3

#### Habituation-dishabituation

Compared to long day mice, exposure to short days did not affect time spent investigating water odor across trials (F_1,16_ = 0.146; *p* = 0.70); however, SD exposure reduced time spent investigating male urine (F_1,16_ = 4.358; *p*≤0.05: Figure 5A). Follow up within-trial *t*-tests revealed that SD mice did not differ from LD mice during the initial exposure to male urine (*t*_16_ = 1.053; *p* = 0.31). SD mice did not spend less time investigating male urine during trial 2 (*t*_16_ = 1.892, *p* = 0.08), but SD mice spent significantly less time investigating during trial 3 (*t*_16_ = 2.452, *p*≤0.05: Figure 5A). There were no differences due to photoperiod in latency to approach the odor source (F_1,16_ = 1.347; *p* = 0.26) (Figure 5B).

## Discussion

In the current study, adult male white-footed mice exposed to short days did not alter measurements of neuronal morphology of the granule cells in the olfactory bulb (Figure 4), or alter proliferation of BrdU+ progenitor cells in the olfactory bulb (Figure 3), which are mostly comprised of neurons (Table 1). However, SD exposure increased BrdU+/NeuN+ cells 4 weeks after injections in the anterior plexiform and granule layers of the olfactory bulbs (Figure 3F,H). Concurrent with an increase in neurogenesis in the caudal olfactory bulbs, SD mice also habituate faster (reduced investigation time) to conspecific male urine odors (Figure 5). These findings demonstrate that, in a photoperiodic rodent, day length can affect both olfactory-mediated behavior and olfactory bulb neurogenesis, which may underlie olfactory learning.

Neural progenitor cells arising from the subventricular zone (SVZ) of the lateral ventricles migrate via the rostral migratory stream (RMS) to the olfactory bulbs, and the caudal olfactory bulbs are closest to the SVZ origin of the proliferating cells, which may account for the differences found in the caudal olfactory bulb in the current study. However, BrdU+ cell numbers 4 weeks after injection did not differ across the rostro-caudal extent of the olfactory bulb (Figure 3), which argues against this possibility. It is also possible that subtle SD increases in SVZ proliferation not detected in this study, altered survival during the migratory process along the RMS from the SVZ to the OB [24], altered programmed cell death of OB progenitor cells [25] in LD mice, or some combination of these factors, contributes to the current findings. Subtle photoperiodic differences in olfactory bulb cell death in *Sorex* shrews, identified by TUNEL labeling, have been reported [17], although overall rates were very low. The influence of photoperiod on these factors in this species remains uninvestigated, thus, we cannot rule out the possibility that subtle alterations in proliferation or progenitor migratory survival, not detected in the current study, contribute to the increase in olfactory bulb neuron survival found in SD mice.

Neuronal precursors arising from the SVZ, or from a pool of quiescent progenitor cells in the central OB [26]–[27], continuously replace a population of the inhibitory interneurons (granular and periglomerular) of the adult olfactory bulb. Two recent studies ablating SVZ neurogenesis in mice have shown opposing roles for newly born neurons in the olfactory bulb: they may be preferentially involved in formation of odor memories [28], or neurogenesis is uncoupled from olfactory bulb function [29]. Although more research is necessary to parse out the specific role of neurogenesis in olfaction, it is possible that turnover and overproduction of new neurons in the olfactory bulb is critical for optimally encoding new olfactory memories [7], whereas old neurons (arising in perinatal development) are responsible for general olfaction ([25]; reviewed in [30]). However, continuous adult neurogenesis is necessary to support innate olfactory-dependent behavioral responses [6] and olfactory experience modulates the turnover of OB olfactory bulb new neurons in a spatial and temporal manner [31]. Although the role of olfactory bulb neurogenesis remains unclear, taken together, a growing body of evidence, including the current study, supports the necessity of neurogenesis for behavioral and physiological plasticity within the olfactory bulb.

Within the olfactory bulbs, odors are topographically represented by glomeruli [32]–[33], and odor-specific activation of patterns of glomeruli allow for differential threshold sensitivity [34] and encoding of olfactory memories by the cortex [35]. Additionally, water-soluble odorants preferentially activate glomeruli in the posterior olfactory bulb [36]. In the current study, SD exposure increased neurogenesis in the posterior olfactory bulb (Figure 3H), and in accordance with the topography of water-soluble odors, SD mice had altered responsiveness to conspecific male urine (Figure 5A). Reduced investigation time of urine may not be a function of short-term habituation, but could potentially reflect altered motivation not related to the odor [37]. However, the current data argue against this possibility because SD mice did not differ from their LD counterparts in latency to investigate all odor presentations (Figure 5B) or investigation behavior of a socially neutral water-soluble odorant (water; Figure 5A).

One of the hallmarks of photoperiodic rodents is day length-dependent plasticity of neural systems, including neuroendocrine and behavioral circuits (reviewed in [11]). Photoperiodic responses in some rodent species are coupled to the olfactory system. In nonphotoperiodic rodents, olfactory bulbectomy (OBX) can unmask photoperiodic responsiveness, as demonstrated in both lab rats (*Rattus norvegicus*; [38]) and house mice (*Mus musculus*; [39]). Olfactory input is necessary for normal photoperiodic responses in gray mouse lemurs [40] and Syrian hamsters [41]–[42], potentially due to OBX-dependent increases in gonadotropins. However, OBX in Siberian hamsters has no affect on photoperiodic responses [43]. Thus, the exact role of olfaction and the olfactory bulbs in photoperiodic responses is species-specific, and in white-footed mice remains uninvestigated.

In common with the current study, seasonal differences in odor responsiveness have been reported for many mammalian species, ranging from blind mole rats [9], to meadow voles [8], [44], to humans [45]. Altered responses to presentation of male urine discovered in the current study may be related to photoperiodic changes in social structure in white-footed mice. During the breeding season, male white-footed mice are territorial and aggressive toward intruding males, whereas exposure to short days facilitates prosocial behaviors as mice form communal groups [46], most likely for thermal energetic conservation. Thus, the facilitation of habituation to conspecific odors we report here in SD mice may support these prosocial behaviors.

In addition to altering behavioral circuits, exposure to short days in photoperiodic rodents, including white-footed mice, alters neuroendocrine and reproductive circuits; reducing paired testes mass (Figure 1) and concentrations of sex steroids and gonadotropins (reviewed in [11]). Although adult neurogenesis has been shown to be modulated by sex steroids [47]–[48], the effects are temporally-, sex-, and species-specific (reviewed in [49]; but see [16], [50]). Additionally, neurogenesis in the SGZ and the SVZ are regulated by different mechanisms [51], and regulation of neurogenesis in some photoperiodic rodents is mechanistically different between SD and LD animals [15], [17]. In common with Syrian hamsters [15], SD white-footed mice in the current study displayed increased neurogenesis in the olfactory bulb (Figure 3H). In contrast, photoperiod does not affect SGZ neurogenesis in Eastern Gray squirrels [50], and SD decreases SGZ and SVZ proliferation and neurogenesis in two species of photoperiodic shrews [17]. Thus, we cannot make generalizations about the interaction of steroids and photoperiod in the regulation of adult neurogenesis, as this needs to be addressed in species-specific manner.

In summary, the current study demonstrates that in white-footed mice, photoperiod alters neurogenesis in the olfactory bulb, without affecting proliferation or granule cell neuronal morphology. The short-day increase in neurogenesis within the caudal olfactory bulb, the region known to respond to water-soluble odorants, is associated with altered behavioral responses to conspecific male urine. These changes in behavior associated with altered olfactory bulb neurogenesis may represent a neural substrate responsible, in part, for photoperiodic changes in social structure in this species. In addition, the current study adds to the growing body of literature describing the role of olfactory bulb neurogenesis in olfactory behaviors, and provides novel insight into the role of photoperiod in the regulation of olfactory bulb-dependent plasticity.
